# Survivin is an independent predictor of short-term survival in poor prognostic breast cancer patients

**DOI:** 10.1038/sj.bjc.6603616

**Published:** 2007-02-06

**Authors:** A R Hinnis, J C A Luckett, R A Walker

**Affiliations:** 1 Department of Cancer Studies & Molecular Medicine, University of Leicester, Leicester Royal Infirmary, Leicester, LE2 7LX, UK

**Keywords:** survivin, breast cancer, prognosis, apoptosis, cell cycle

## Abstract

Established clinico–pathological factors can place patients with breast cancer into good and poor prognostic categories, but even within these groups behaviour and response to treatment can differ. This study examined the value of cell cycle and apoptotic regulatory proteins in predicting behaviour in a poor prognostic group. A total of 165 patients, all of whom had died of breast cancer with duration of survival 12–127 months, median 38 months, were examined using immunohistochemistry for proliferation, apoptosis, p53, phosphorylated p53, p21, checkpoint kinase 2 (Chk2), bcl-2, bax, survivin and XIAP. All had received chemotherapy and/or hormonal therapy and were predominantly T2, node positive, grade III with only half oestrogen-receptor (ER) positive. High proliferation, phosphorylated p53, Chk2 and survivin expression correlated with grade III and lack of ER, whereas low proliferation, p21 and bcl-2 related to better grade and presence of ER. On univariate analysis grade, proliferation, phosphorylated p53, bcl-2, ER and survivin related to duration of survival. In multivariate analysis, grade (*P*=0.001) and survivin (*P*=0.005) were independent prognostic factors, grade III and presence of survivin relating to shorter survival. The latter was particularly for those patients receiving neoadjuvant therapy and adjuvant chemo- and hormonal therapy. The presence of the inhibitor of apoptosis protein survivin is a highly significant independent predictor of shorter duration of survival of patients with poor prognostic features, and merits investigation as a marker in other prognostic groups.

It is well recognised that breast cancer is a heterogeneous disease. Although there has been an improvement in outcome owing to better early detection and the use of adjuvant therapy (Early Breast Cancer Trialists Collaborative Group, 2005), behaviour is variable. For the management of individual patients there is a need to have markers that can identify those cancers likely to have a better or a worse prognosis (prognostic) and also aid the selection of appropriate therapy (predictive) (Gelber *et al*, 2003; Hayes, 2003). Clinico-pathological factors such as tumour size, node status and tumour grade are acknowledged important prognostic factors (Carter *et al*, 1989; Elston and Ellis, 1991; Henson *et al*, 1991; Galea *et al*, 1992; Gebauer *et al*, 2002). Oestrogen receptor (ER) status has been used for many years for the selection of those patients who are more likely to benefit from hormone treatment, either for metastatic disease (Elledge *et al*, 2000) or as adjuvant therapy (Harvey *et al*, 1999). There are no comparable predictive markers for identifying those patients for whom chemotherapy will or will not be effective.

The *TP53* gene has important roles in cell cycle regulation and apoptosis following DNA damage (Harris, 2000; Vousden and Lu, 2002). The effect of several chemotherapeutic drugs may be mediated through DNA damage and induction of apoptosis (Gewirtz, 2000), and therefore alterations to *TP53* and hence p53 protein function could result in relative resistance to these agents. Studies assessing either immunohistochemically detectable p53 and/or *TP53* mutations in breast cancers and response to chemotherapy differ in their findings as to their value as predictors (Bergh *et al*, 1995; Elledge *et al*, 1995; Stal *et al*, 1995; Aas *et al*, 1996; Thor *et al*, 1998; Berns *et al*, 2000; Rahko *et al*, 2003). However, as apoptosis plays a major role in treatment response, analysis of other apoptotic regulatory gene products, as well as p53, may give additional information.

The bcl-2 family of apoptotic proteins comprise anti-apoptotic proteins such as bcl-2 and bcl-xl, and pro-apoptotic proteins such as bax, and these form heterodimers (Reed, 2000). P53 promotes apoptosis by inducing bax, so increasing the ratio of pro- to anti-apoptotic proteins (Haupt *et al*, 2003). The presence of bcl-2 in breast cancers positively correlates with ER (Gee *et al*, 1994). *In vitro* drug response assays showed Bcl-2-negative breast cancers to be more susceptible to chemotherapeutic drugs than bcl-2-positive (Yang *et al*, 2000), but immunohistochemical studies of breast cancers have been less conclusive (Krajewski *et al*, 1995). Although one study found reduced expression of bax to be associated with a poor response to chemotherapy in patients with metastatic breast cancer (Daidone *et al*, 1999a), others have not (Daidone *et al*, 1999b; Sjöstrum *et al*, 2002). The inhibitors of apoptotic proteins are a more recently described family of proteins that includes survivin and XIAP, which act by direct inhibition of caspases (Deveraux and Reed, 1999). Survivin expression is negatively regulated by wild-type p53 and it participates in p53-dependent apoptotic pathways (Mirza *et al*, 2002). One study has shown that the presence of survivin in breast cancer relates to apoptosis but not prognosis (Tanaka *et al*, 2000). Another found it to relate to poor prognosis (Span *et al*, 2004) and one (Kennedy *et al*, 2003) to a better prognosis. There are little data about XIAP in breast. Parton *et al* (2002) reported that XIAP was not a predictive marker of response to chemotherapy, but the small sample size may not have been representative.

P53 is also involved in cell cycle arrest and induces p21^waf1^ in response to DNA-damaging agents (el-Deiry *et al*, 1994). p21 blocks progression of the cell cycle at G1/S and is involved in G2/M phase arrest (Dulic *et al*, 1998). Caffo *et al* (1996) concluded from a study of patients who received adjuvant therapy and had long-term follow-up that p21−/p53+ tumours had the worst prognosis and that this pattern of expression may be of relevance for therapeutic response. However, Sjostrum *et al* (2000) found no relationship between expression of p21 and p53 and response to chemotherapy in advanced disease. Checkpoint kinase 2 is a cell cycle checkpoint protein that can regulate p53 by phosphorylation in response to DNA double-strand breaks (Bartok and Lukas, 2004). Loss of function of Chk2, by downregulation of expression and p53 by mutation, has been reported in a proportion of sporadic breast cancers (Sullivan *et al*, 2002). A recent immunohistochemical study showed reduction of Chk2 expression to relate to larger tumour size but not to other parameters (Kilpivaara *et al*, 2005).

Even within patients with cancers having similar poor prognostic features there can be differences in duration of survival. Our hypothesis was that p53, apoptotic and cell cycle regulatory proteins in combination would provide greater information about breast cancer behaviour. This was tested in a group of patients who had received chemotherapy and/or endocrine therapy, who had a higher incidence of poor prognostic features at presentation and who had died of the disease. Immunohistochemistry was the method of choice, as this tool is widely available and used clinically for predictive testing.

## MATERIALS AND METHODS

### Patients

A total of 165 patients diagnosed with breast cancer at University Hospitals of Leicester NHS Trust between 1991 and 2002 were studied and all had died from the disease. A breast cancer patient database, established by Miss A Stotter, was used to identify patients' complete data for all patients, treated by two surgeons. The complete data was available for 1991 and 1997–2002 and the incomplete data was available for 1992–1996. All patients who had received chemotherapy and for which sufficient tissue was available were included. Patients receiving only tamoxifen were restricted to those 70 years and under and were selected to represent a wide range of survival duration. All information on treatment and cause of death had been validated. A total of 18 patients had received neoadjuvant chemotherapy before surgery, with nine having subsequent adjuvant hormonal therapy. All had residual tumour present in the subsequent mastectomy, with eight having a reduction in tumour size. A total of 147 patients had either mastectomy or wide local excision with axillary surgery followed by adjuvant therapy: Eighteen chemotherapy alone; 70 chemotherapy followed by Tamoxifen, 59 Tamoxifen alone. Chemotherapy was cyclophosphamide, adriamycin and fluorourocil or cyclophosphamide, methotrexate and fluorourocil. Patient ages at diagnosis ranged from 22 to 70 years, median 53 years. For analysis they were grouped as 22–48 years (premenopausal), 49–56 years (perimenopausal) and 57–70 years (postmenopausal). Survival was from 12 to 127 months with median for the whole group of 38 months. Median survival for treatment groups are shown in Table 1.

### Tissues

All tissues relating to the patients were then identified from the files of the histopathology department, University Hospitals of Leicester NHS Trust. For those patients receiving neoadjuvant chemotherapy needle core biopsies were available for six and these were used for analysis, as well as the surgical material. All surgical specimens had been received fresh, sliced and fixed for 24 h in 4% formaldehyde in saline. Blocks had been selected by the pathologist, processed through graded alcohols and xylene and embedded in paraffin wax.

Information was available from pathology reports for tumour size and lymph node status. All tumours were classified and graded by RA Walker using NHS Breast Screening Programme pathology reporting guidelines (NHSBSP, 2005). Oestrogen receptor-*α* status and HER-2 status was assessed using immunohistochemistry, and reported by RA Walker (Walker *et al*, 1996). The research project was approved by Leicestershire Research Ethics Committee (Ref 7117).

### Antibodies

p53: D07 mouse monoclonal antibody specific for human p53 protein wild-type and mutant forms; NCL-p53-PHOS mouse monoclonal antibody that detects human p53 protein phosphorylated at Ser^392^ (both Novocastra, Newcastle, UK).

Bax polyclonal rabbit anti-human bax antiserum, raised against amino acids 43–61 (Dako, Ely, UK) polyclonal rabbit anti-human bax antiserum raised against amino acids 150–165 (Oncogene, Cambridge, USA)

bcl-2: NCL-bcl-2 mouse monoclonal antibody (Novocastra, UK). bcl-2 Ab-1 mouse monoclonal antibody raised against amino acids 41–54 (Oncogene, Newcastle).

p21^waf1^: NCL-WAF1 mouse monoclonal antibody raised against full-length p21 (Novocastra, UK).

Chk2: NCL-Chk2 mouse monoclonal antibody raised against full-length Chk2 protein (Novocastra, UK).

Ki67: MIB1 mouse monoclonal antibody raised against recombinant Ki-67 (Novocastra, UK).

Survivin: Ab469 rabbit polyclonal antiserum raised against full-length human survivin protein (Abcam Ltd, Cambridge, UK)

XIAP: Goat polyclonal antiserum raised against amino acids 1–497 of human XIAP (R&D, Abingdon, UK).

M30: Cyto DEATH mouse monoclonal antibody against caspase cleaved cytokeratin 18 (Roche, Lewes, UK)

Secondary reagents: Biotinylated anti-mouse, rabbit and goat immunoglobulin antibodies, streptavidin–biotin complex (all DAKO, UK); peroxidase-labelled anti-rabbit and mouse immunoglobulin antibodies (Amersham, UK); peroxidase labelled anti-goat immunoglobulin antibody (Calbiochem, Nottingham, UK)

### Immunohistochemistry

Sections were put onto Vectabond (Vector, Peterborough, UK) oated glass slides and dried. Endogenous peroxidase was blocked with 2% hydrogen peroxide in water for 10 min, apart from sections to be stained for survivin and XIAP. Antigen retrieval was in 0.2 M citrate buffer (pH 6.0) with pressure cooking for 4 min for XIAP, 2 min followed by 2 min for survivin and 2 min for all other antigens.

Primary antibodies were applied for 18 h at 4°C. Optimised dilutions were: p53 D07 1 : 50; p53 PHOS 1.25; p21^waf1^ 1 : 20; ChK2 1 : 40; bcl-2 (Novocastra) 1 : 80; bax (DAKO) 1 : 50; MIB-1 1 : 100; M30 1 : 200; survivin 1 : 400; XIAP 1 : 800. All dilutions and washes were in Tris-buffered saline (pH 7.6) apart from for M30, which was phosphate-buffered saline with 1% bovine serum albumin and 0.1% Tween 20. Appropriate biotinylated secondary antibodies were applied for 30 min at room temperature followed by streptavidin–biotin–peroxidase complex. Peroxidase was localised using diaminobenzidine, with counterstaining with Mayers haematoxyin. Negative controls were omission of primary antibody. A known positive control was used with each batch of staining.

For all nuclear staining the number of positive cells were counted in 1000 cells in five high-power fields (hpf) and percentage derived. For M30 the number of positive cells in 10 hpf was also counted. Cut-off points were: p53 and p53 PHOS – moderate or strong staining ⩾20% cells = positive; p21^waf1^ ⩾5% cells staining = positive; Chk2 ⩾20% cells staining = positive; MIB1<20% cells staining, low proliferation index, ⩾20%, high proliferation index.

For bcl-2 the percentage of positive cells was determined as 0 (0–4%), 1 (5–25%), 2 (26–50%), 3 (51–75%), 4 (76–100%) and combined with intensity 1 (faint), 2 (moderate), 3 (strong) relative to lymphocytes. Score of 0–2 was considered negative.

For bax staining, intensity was assessed as negative, faint, moderate or strong, with moderate and strong classed as positive.

Survivin was assessed in 5 hpf. Nuclear staining was scored as 0 (<5%), 1 (5–20%), 2 (21–50%), 3 (⩾50%), cytoplasmic as 0 (negative or faint), 1 (moderate), 2 (strong) then scores were combined with ⩽1 being considered negative (33). XIAP was assessed for intensity as 1 (negative or faint), 2 moderate, 3 (strong), with 2 and 3 being positive.

### Statistics

SPSS version 12.0 for Windows was used and data variables were entered either as continuous or as grouping variables. *χ*^2^ test was used to compare two or more grouping variables. *t*-test was used to compare two groups against a continuous variable and one-way analysis of variance for three or more groups against a continuous variable. Kruskall–Wallis test was used for nonparametic data. Log rank Kaplan–Meier test was employed to assess relationships to duration of survival and Cox regression analysis was used to determine strongest predictive factors.

## RESULTS

### Clinico–pathological parameters, ER and HER-2

The distribution of duration of survival, age of patient, tumour type, size, node status and grade in relation to the different treatment groups are shown in Table 1. There was a higher frequency of postmenopausal patients receiving adjuvant hormonal therapy. The majority of cases were infiltrating ductal carcinoma NST with three quarters being T2 or greater, node positive and grade III.

Over half of the cancers were ER-positive. There was a significant correlation with grade, with a higher frequency of grade III cancers being negative (*P*<0.0001). About 25% of cases were HER-2-positive. There was an inverse correlation with ER status (*P*=0.01), but no relationship to tumour size, node status or grade.

### Proliferation and cell cycle proteins

A high proliferation index (⩾20% cells staining for Ki-67) was found in 58.2% of cancers, with a significant correlation between high index and grade III (*P*<0.0001) but not with other clinico–pathological parameters. There was an inverse correlation with ER (*P*=0.001).

Staining for p21^waf−1^ was predominantly nuclear and only this was assessed. A total of 27.3% of cancers were positive with a ⩾5% cutoff. Grade III tumours were more likely to be negative (96/123) (*P*=0.015). There was a correlation between the presence of p21^waf−1^ and ER (*P*=0.015). There was no relationship with proliferation.

ChK-2 protein was detected in ⩾20% of tumour cells in 69.7% of cancers and showed a significant correlation with grade III cancers (*P*=0.003) and high proliferation (*P*=0.005). There was an inverse relationship with ER (*P*=0.012).

### p53

p53 protein was detected in 62/165 breast cancers (37.6%) and phosphospecific p53 (phosphorylated at Ser^392^) in 22.4%. There was a strong correlation (*P*<0.0001) between them, with all phosphospecific p53 cancers being positive with the D0–7 antibody. The presence of phosphospecific p53 correlated with grade III (*P*=0.002) but there were no other correlations with clinico–pathological parameters for p53 or phosphospecific p53. There was an inverse relationship between the presence of phosphospecific p53 and ER (*P*<0.0001), but no relationship with HER-2. Carcinomas with detectable cp53 and phosphospecific p53 were more likely to have a high proliferation index (*P*=0.001).

### Apoptosis and apoptotic proteins

Cells in early apoptosis were characterised by cytoplasmic staining with M30 and those in advanced stages were shrunken with dark staining cytoplasm. When the percentage of apoptotic cells was calculated, there were 55% of tumours with <1%, 24% with 1–2% and 21% with >2%. For assessment of 10 hpf there were 37% of cancers with <10, 33% with 10–20 and 30% with >20. There was no correlation between apoptotic index and any clinico–pathological parameter, ER, HER-2, p53, cell cycle protein or apoptotic protein.

Nearly half of the cancers (49.7%) were bcl-2-positive. There was a significant correlation between lack of bcl-2 and grade III (*P*<0.0001) and the presence of bcl-2 and ER (*P*<0.0001). Bcl-2-positive carcinomas were more likely to have lower proliferation (*P*=0.001). There was a weak association between lack of bcl-2 and presence of HER-2 (*P*=0.05).

Homogenous cytoplasmic staining of varying intensity was seen for bax, with 91.0% of cases considered positive. There were no correlations with clinico–pathological parameters, ER or HER-2.

Survivin was detected in cancer cells either in the nucleus, nucleus and cytoplasm or cytoplasm, with the combined pattern of staining predominating. Staining of both components was assessed and combined. A total of 55.2% cancers were considered positive. There was a significant correlation between the presence of survivin and grade III (*P*<0.0001) and high proliferation (Ki-67)(*P*<0.0001) with an inverse correlation with ER (*P*<0.0001). There was a weaker correlation between the presence of survivin and HER-2 positivity (*P*=0.02).

XIAP was detected as homogenous cytoplasmic staining with variation in intensity. Those cases with moderate or strong staining were considered positive (89.7%) but there were no correlations with clinico–pathological parameters, ER and HER-2.

### Correlations between markers

Table 2 summarises those markers for which there were significant correlations. The main findings were the positive correlations between p53 and the IAP family members and the inverse relationships between bcl-2 and p53 and bcl-2 and survivin.

### Relationships to survival

This was considered for the tumours overall and in relation to the treatment received.

There was a significant correlation between duration of survival and tumour grade for patients receiving adjuvant treatment (*P*<0.0001), with grade III tumours having a shorter duration of survival. Within the group receiving hormone therapy alone, smaller size was associated with longer survival (*P*=0.005), but overall size did not relate to duration of survival and neither did node status. The presence of ER was associated with longer duration of survival but only for the group receiving adjuvant hormonal treatment alone (*P*<0.0001). There was no relationship between HER-2 status and duration of survival.

In univariate analysis the factors that related to duration of survival overall were overall tumour grade (*P*⩽0.0001), ER (*P*⩽0.001), proliferation (*P*=0.001), phosphorylated p53 (*P*⩽0.0001), bcl-2 (*P*=0.01) and survivin (*P*⩽0.0001). For those receiving neoadjuvant chemotherapy the presence of survivin related to shorter duration of survival (24 *vs* 48 months for negative cases) (*P*⩽0.0001). In the adjuvant therapy group grade was significant with grade III cancers being associated with a shorter duration of survival. Smaller size related to longer duration of survival for those receiving hormonal therapy (*P*=0.005) but size had no effect on other groups. This was similar for ER, bcl-2 and p21, where presence related to longer duration only for the group receiving adjuvant hormonal treatment (*P*<0.0001, 0.039 and 0.014, respectively). Those patients whose cancers were positive for phosphorylated p53 and who received either adjuvant hormonal or adjuvant chemo-plus hormonal therapy had a significantly shorter duration of survival (*P*=0.003 and 0.026 respectively). The presence of survivin related to shorter duration of survival (32 months in comparison to 55 months for those negative) for the group receiving adjuvant chemotherapy and hormonal therapy (*P*⩽0.0001).

A Cox regression model analysis of the factors found on univariate analysis to be associated with duration of survival showed that overall tumour grade and survivin were the most significant (Table 3). When this was undertaken in relation to treatment received, survivin was a significant factor for the neoadjuvant and adjuvant chemotherapy and hormonal therapy groups and ER for the adjuvant hormonal therapy group.

## DISCUSSION

The aim of this study was to determine whether analysis of apoptotic and cell cycle regulatory proteins could provide information about the behaviour of poor prognostic breast cancers, particularly in relation to the type of therapy used. Induction of apoptosis and decreased proliferation are factors in the biological response of breast cancer to chemotherapy and endocrine therapy (Dowsett *et al*, 1999) and, therefore, defects in their regulatory machinery could relate to poor response. As well as examining proteins more extensively studied, such as p53, bcl-2, bax and p21, the phosphorylation status of p53, Chk2 and the IAP family members survivin and XIAP were considered.

The patients had all died of breast cancer, were of a lower median age (National Institutes of Health Consensus Development Panel, 2001), with a high frequency of T2 and above, node positive, grade III cancers. They therefore differ from many early stage breast cancer patients but are still an important clinical group and can provide information of relevance to other breast cancer patients.

The most significant finding related to the IAP protein, survivin. The presence of survivin had a highly significant relationship with shorter duration of survival in both univariate and multivariate analysis, particularly for the neoadjuvant therapy and adjuvant chemotherapy plus hormonal therapy groups. A previous study by Span *et al* (2004), which measured Survivin mRNA, found expression to be an independent marker of relapse free and overall survival although another examining mRNA did not (O'Driscoll *et al*, 2003). Span *et al* (2004) found similar relationships between survivin, grade III and lack of ER. A recent report in which enzyme-linked immunosorbent assay was used to measure survivin found high levels to relate to poorer outcome, which was independent of other factors (Ryan *et al*, 2006). However, two immunohistochemical studies report different findings to the present one. Tanaka *et al* (2000) found no relationship between survivin and p53 or ER, but a positive correlation with bcl-2 and reduced apoptotic index. The latter correlated to survival but survivin did not. A different antibody was used which only gave cytoplasmic reactivity. The numbers studied were similar with many being T2 and 61% node positive. Kennedy *et al* (2003) used the same antibody (AB469) as the present study but found a correlation between presence of Survivin and better prognosis. They evaluated nuclear and cytoplasmic reactivity separately and found nuclear staining to be associated with better outcome, whereas cytoplasmic staining was not significant. We found that the majority of tumours that expressed survivin had a combined pattern of reactivity and therefore did not undertake separate analysis. In some tumours, survivin was only observed in cells undergoing mitosis. The ratio of detection of cytoplasmic to nuclear reactivity can vary depending on the antibodies used (Fortugno *et al*, 2001). A recent review of the significance of survivin in cancers generally (Li *et al*, 2005) identified more studies in which it was found to relate to poorer prognosis, and recommended that for technical reasons nuclear and cytoplasmic staining should not be considered separately, the approach used in the present study.

Although survivin was originally considered to be an inhibitor of apoptosis (Deveraux and Reed, 1999) it has now been implicated in cell proliferation, having functions at cell division to control microtubule stability and mitotic spindle assembly (Fortugno *et al*, 2001; Giodini *et al*, 2002; Beardsmore *et al*, 2004). Our findings that there was no relationship between survivin and apoptosis and an inverse relationship with the anti-apoptotic protein bcl-2, plus a correlation with high grade and high proliferation indicates that the function of survivin within the carcinomas studied relates to its role in cell proliferation rather than apoptosis.

A possible correlation between expression of HER-2 and survivin has been reported (Asanuma *et al*, 2005) but was not found in the present study. The frequency of HER-2-positive cases was quite different, 66% compared to 25% in the present study that would be expected for such a patient group, which may explain the difference in relationships.

Immunohistochemical evaluation of p53 was of no value. There are several problems in using this technique for p53 that relate to a failure to detect all mutations plus assessment of what is positive (Hall and McCluggage, 2006) so the available evidence would favour the use of mutational analysis (Bergh *et al*, 1995; Aas *et al*, 1996; Berns *et al*, 2000). Phosphorylation of p53 at Ser^392^ has been shown to be a frequent modification in tumours (Minamoto *et al*, 2001). The biological role of ser^392^ phosphorylation of wild-type p53 is unclear, but it may regulate the oncogenic function of mutant p53 (Yap *et al*, 2004). Immunohistochemical detection of phosphorylated Ser^392^ is more frequent in breast cancers with mutant rather than wild-type p53 (Nenutil *et al*, 2005). In other tumours the presence of p53 phosphorylated at Ser^392^ has been associated with poorer survival (Matsumoto *et al*, 2004). In this series its presence related to a shorter duration of survival, particularly for those patients receiving adjuvant hormonal therapy. It is recognised that *TP53* mutations can predict poor response to tamoxifen (Berns *et al*, 2000). Phosphorylation of Ser^15^ has also been detected in breast cancers with mutant p53 (Nenutil *et al*, 2005) and would merit further analysis.

Reduced expression of ChK2 has been reported in breast cancer with and without mutations (Sullivan *et al*, 2002; Kilpivaara *et al*, 2005), with similar correlations to tumour characteristics as in the present study, but with no relationship to survival. Mutation status may be more important as a marker of predisposition than protein loss and tumour behaviour.

The findings for bcl-2 are similar to those reported by others (Gee *et al*, 1994; Daidone *et al*, 1999a) with correlations to better grade, presence of ER and longer survival. Unlike others (Krajewski *et al*, 1995) bax did not contribute. The presence of p21 was associated with markers of a better prognosis and longer duration of survival for those receiving adjuvant hormonal therapy, which differs from that of Caffo *et al* (1996), who found it to be associated with shorter duration of survival, whereas Gohring *et al* (2001) found no relationship. Oestrogen receptor related to longer duration of survival but this was for the group receiving hormonal therapy, emphasising that any impact it has on survival relates to its value as a predictive marker of response. The study also demonstrates that grade, when carefully performed following guidelines, is an important independent prognostic indicator of value for all breast cancers.

In conclusion, the inhibitor of apoptosis protein survivin is a highly significant predictor of shorter duration of survival in patients with poor prognostic breast cancer and merits evaluation in a wider group of patients.

## Figures and Tables

**Table 1:** Treatment regime

	**Neo Adjuvant**	**Adjuvant Chemo**	**Adjuvant Chemo+Hormonal**	**Hormonal**	**Total**
**Type**	**No (%)**	**No (%)**	**No (%)**	**No (%)**	**No (%)**
IDC	18 (100)	18 (100)	63 (90)	58 (98.3)	157 (92.2)
ILC	0	0	7 (10)	0	7 (42)
Others	0	0	0	1 (17)	1 (0.6)
					
*Size*					
<20 mm	5 (25.5)	4 (2.2)	12 (7.9)	20 (33.9)	40 (24.2)
20–50 mm	12 (67.0)	13 (72.2)	51 (76.1)	36 (61.0)	112 (67.8)
>50 mm	1 (5.5)	1 (5.6)	4 (6.0)	3 (5.1)	9 (5.4)
					
*Grade*					
I+II	2 (11.1)	1 (5.6)	14 (20.3)	24 (40.7)	41 (25.0)
III	16 (88.9)	17 (94.4)	55 (79.7)	35 (59.3)	123 (75.0)
					
*Lymph node*					
Neg	4 (23.5)	5 (27.8)	7 (10.3)	26 (44.8)	42 (26.0)
1–3	6 (35.3)	7 (38.9)	24 (35.3)	19 (32.8)	56 (34.7)
>3	7 (41.2)	6 (33.3)	37 (54.4)	13 (22.4)	63 (39.1)
					
*ER*					
Neg	9 (50.0)	8 (44.4)	33 (47.8)	30 (50.8)	80 (48.7)
Pos	9 (50.0)	10 (55.6)	36 (52.2)	29 (49.2)	84 (51.2)
					
*Age*					
22–48	11	15	25	2	53
49–56	3	2	27	19	51
57–70	4	1	18	38	61
Duration of survival median (months)	32	43	33	56	38

IDC=invasive ductal carcinoma; ILC= invasive lobular carcinoma

**Table 2:** Correlation between p53, cell cycle and apoptotic proteins (+=positive; −=negative)

**Marker v marker**	***P*-value**
p53 + bcl-2 −	0.005
phospho p53 + bcl-2 −	0.004
p53 + survivin +	<0.0001
Phospho 53 + survivin +	<0.0001
p53 + XIAP +	0.02
Phospho p53 + p21^waf1^ −	0.033
ChK2 + survivin +	0.003
bcl-2 − survivin +	0.001
	
	

ChK2=checkpoint kinase 2

**Table 3:** Cox regression analysis of factors in relation to duration of survival

	**B**	**SE**	**Wald**	**Sig**
p53 phos	0.0018	0.309	0.003	0.953
MIB1	0.134	0.294	0.201	0.649
bcl-2	0.338	0.296	1.305	0.253
Survivin	0.693	0.248	7.827	0.005
Grade	1.477	0.397	13.845	0.000
Size	0.228	0.160	2.037	0.153
ER	0.014	0.408	0.001	0.974

ER=oestrogen receptor
